# Effect of Vitamin D supplementation on body composition and cardiorespiratory fitness in overweight men—a randomized controlled trial

**DOI:** 10.1007/s12020-018-1665-6

**Published:** 2018-07-05

**Authors:** Christos Karefylakis, Stefan Särnblad, Annaclara Ariander, Gustaf Ehlersson, Eva Rask, Peter Rask

**Affiliations:** 1 Department of Endocrinology, School of Medical Sciences, Örebro University, SE 70182 Örebro, Sweden; 2 Department of Pediatrics, School of Medical Sciences, Örebro University, SE 70182 Örebro, Sweden; 3 School of Medical Sciences, Örebro University, SE 70182 Örebro, Sweden; 4 Department of Clinical Physiology, Örebro University Hospital, SE 70185 Örebro, Sweden

**Keywords:** Vitamin D, Body composition, Cardiorespiratory fitness, Bioelectrical impedance analysis, Cardiopulmonary exercise test

## Abstract

**Purpose:**

Several observational studies have shown an association between vitamin D deficiency and non-skeletal major health issues including impaired cardiorespiratory fitness and adiposity. Only a few studies have examined the impact of vitamin D supplementation on these conditions and the results are ambiguous. The aim of this study was to examine the effect of vitamin D supplementation on body composition and cardiorespiratory fitness in overweight/obese men with vitamin D deficiency.

**Methods:**

This study was a prospective, placebo controlled, double blinded, randomized trial with a study period of 6 months. Forty overweight/obese men (BMI > 25 kg/m^2^) with vitamin D deficiency (25(OH)D ≤ 55 nmol/L) were randomized to receive either 2000 IU Cholecalciferol drops or the equivalent amount of drops of placebo. At baseline and follow up body composition and cardiorespiratory fitness were measured and blood samples were obtained. Body composition was measured using bioelectrical impedance analysis (BIA) and cardiorespiratory fitness using cardiopulmonary exercise test (CPET). The primary outcomes were changes in percentage body fat and in maximum oxygen uptake (VO_2max_).

**Results:**

No statistically significant difference between the placebo and the intervention group regarding changes in percentage body fat (*p* = 0.54) and VO_2max_ (*p* = 0.90) was observed. Moreover, there was no statistically significant difference between the groups concerning changes in BMI (*p* = 0.26), maximum load (*p* = 0.89) and oxygen uptake at anaerobic threshold (AT) (*p* = 0.14).

**Conclusions:**

We conclude that treatment with 2000 IU/d vitamin D for 6 months does not impact body composition or maximum oxygen uptake in overweight/obese men with vitamin D deficiency.

## Introduction

Vitamin D deficiency has long been associated with poor skeletal health and diseases such as osteomalacia and osteoporosis [1]. Recently, several observational studies have shown associations between low vitamin D status and non-skeletal major health issues including increased risk of cardiovascular diseases [2], diabetes mellitus [3], impaired physical functioning [4] and increased all-cause mortality [5, 6]. It is estimated that 1000 to 2000 IU/d of vitamin D are necessary to satisfy the body’s need for most people [7] and the tolerable upper intake levels are 4000 IU/d for ages 9 and older [8].

Obesity is a growing global health issue, with a prevalence that has doubled worldwide since 1980. Approximately 39% of adults over 18 years in the world were overweight and 13% obese in 2016 [9]. Overweight is associated with metabolic risk factors and with higher mortality, both all-cause death and cardiovascular related death [2, 10]. It has been consistently found that vitamin D levels are reduced among patients with obesity and an inverse association between vitamin D and adiposity has been confirmed [11]. Many explanations for this association have been proposed including lack of sun exposure, dietary habits, decreased hepatic 25-hydroxylation in the steatotic liver and increased sequestration of 25OHD in adipose tissue [12]. Higher plasma 25-hydroxy-vitamin D (25(OH)D) has been associated with lower amount of visceral and subcutaneous adipose tissue and with reduced omental adipocyte size suggesting a functional link between vitamin D status and fat distribution. This finding is supported by the fact that the vitamin D receptor (VDR) is expressed in adipocytes and is dynamically upregulated during adipogenesis [13]. Moreover, several in vitro studies in mouse and human adipocytes have demonstrated that 1,25(OH)_2_D_3_ inhibits chronic inflammation in adipose tissue [14]. Despite the strong epidemiological associations between 25(OH)D and obesity in cross-sectional studies, treatment with vitamin D does not seem to affect body weight [12] but has been found to decrease insulin resistance [15] and to decrease blood pressure [16]. Data concerning possible effects of vitamin D supplementation on body composition are ambiguous [17, 18].

Low cardiorespiratory fitness, measured as maximal oxygen consumption (VO_2max_), is independently associated with increased cardiovascular and all-cause mortality [19–21]; it has also been associated with low vitamin D levels in numerous cross-sectional studies [22–24]. The presence of the VDR in cardiac muscle, vascular tissue and skeletal muscle supports the hypothesis that vitamin D may impact the cardiovascular system’s ability to transport oxygenated blood and the skeletal muscles’ ability to use oxygen [25]. Vitamin D deficiency is associated with anaerobic type II muscle fiber atrophy in rats, the main fiber type recruited during maximal strength-type exercise [26], and treatment with the vitamin D analog 1-alpha-hydroxycholecalciferol and calcium induced an increase in the relative number of type II A fibers and a reduction of the type II B fibers [27]. Recently, treatment of severe 25(OH)D deficiency has shown to increase the mitochondrial maximal oxidative phosphorylation in skeletal muscle [28]. A few studies have examined the impact of vitamin D supplementation on cardiorespiratory fitness and the results are not unequivocal [29, 30].

The aim of this study was to examine the effect of vitamin D supplementation on body composition and on cardiorespiratory fitness in overweight men with vitamin D deficiency.

## Subjects and methods

### Trial design

This study was a prospective, placebo controlled, double blinded, randomized clinical trial with an allocation ratio of 1:1. The duration of the study was six months.

### Participants

Participants were recruited using flyers in local health centers, at Örebro university hospital, at the University of Örebro and by an advertisement in the local paper.

The inclusion criteria were male gender, age between 18–70 years old, overweight/obese (BMI > 25 kg/m^2^) and vitamin D deficiency defined as 25(OH)D ≤ 50 nmol/L. Due to difficulties with the recruitment of participants we included even nine subjects with 25(OH)D between 50 and 55 nmol/L. We believe that this deviation would not influence our results and could be justified by the fact that there is a variation in the method of vitamin D analysis (intra-assay coefficient of variation: 8.3%, inter-assay coefficient of variation: 4.3%).

The exclusion criteria were heart disease requiring treatment, severe lung disease in need of treatment, on-going treatment with vitamin D, hypercalcemia (albumin-corrected calcium > 2.55 mmol/L), inability to perform the physical fitness test (bicycle) and participation in another intervention trial at the same time.

### Interventions

The individuals who answered the advertisements were initially screened for vitamin D deficiency. If they met the inclusion criteria they were invited to a first visit at the Örebro University Hospital where a medical history was taken and a routine physical examination including measurements of height, weight, and blood pressure took place. The medical history included present and previous diagnoses and focused on cardiovascular disease, hypertension, hyperlipidemia and diabetes mellitus. When measuring weight the participants had light clothing and the weight was rounded off to the nearest 0.1 kg. Height was measured with a wall-mounted stadiometer to the nearest 0.5 cm.

To complement the medical history an initial blood sample was obtained, analyzing parathyroid hormone (PTH), calcium, creatinine, sodium, potassium, lipid levels, glucose, insulin, and hemoglobin. At this first visit the physician also obtained a written informed consent from each participant.

The individuals who met the inclusion but not the exclusion criteria were enrolled in the study and baseline measurements of body composition and cardiorespiratory fitness were performed. The enrolled individuals were then randomized, in a double-blind manner, to either receiving vitamin D drops (Cholecalficerol, ACO Hud Nordic AB, Sweden) or placebo drops for six months. The intervention group received 2000 IU cholecalciferol/day (25 drops, 80 IU/drop); the placebo group received the same amount of rapeseed oil.

Three months from the initial blood sample another blood sample was collected, analyzing calcium and creatinine, to rule out hypercalcemia which would discontinue the participant from the study.

At the follow up visit, after six months, the participants were asked to report if they had changed their physical activity (decreased, unchanged or increased) during these 6 months. The baseline blood tests were repeated and a second measurement of body composition and cardiorespiratory fitness was performed.

To ensure and monitor compliance bottles with study drug were issued at baseline and after 3 months. At three months and 6 months the used bottles were returned to the research nurse who measured the amount of residual.

Sweden is an elongated country located in Northern Europe with the average sunlight hours varying significantly with season. The latitude of Örebro County is 59° N and the average monthly sunlight hours during December are 30 h compared to 260 h in June [31]. The study started in September–November 2015 and ended in April–June 2016 in an attempt to avoid the impact of seasonal variation on vitamin D levels.

### Biochemical analyses

All biochemical analyses were performed at the laboratory of the University Hospital of Orebro. 25(OH)D was measured using a Liquid Chromatography–Tandem Mass Spectrometry system (Waters Acquity UPLC system, Milford, MA, USA). PTH was measured with Cobas e411 (Roche Diagnostics GmbH, Mannheim, Germany), calcium, creatinine, sodium, potassium, cholesterol, high density lipoprotein (HDL), low density lipoprotein (LDL) and glucose with Vitros 5.1 (Ortho Clinical Diagnostics, Raritan, NJ, USA), hemoglobin with Sysmex XE-5000 (Sysmex Corporation, Kobe, Japan) and insulin with Abbott Architect i2000SR (Abbott manufacturing inc., Irving, TX, USA).

### Outcomes

The primary outcomes were changes in percentage body fat and in maximum oxygen uptake (VO_2max_). The secondary outcomes were changes in BMI, maximum load and oxygen uptake at anaerobic threshold (AT).

The body composition was measured using bioelectrical impedance analysis (BIA) (Body composition analyser 420, Tanita). BIA is a relatively simple, quick, portable, and noninvasive method and has been validated for use as measurement of body composition in obese adults [32]. Subjects were measured while standing erect, in bare feet, on the analyzer’s footpads and wearing either light clothing or undergarments.

The cardiorespiratory fitness of the participants was evaluated using cardiopulmonary exercise test (CPET). Initially, a dynamic spirometry was performed in order to receive a flow-volume loop. Then resting oxygen uptake and carbon dioxide production was measured using a sealed facial mask that analyzes breathing gases (Jaeger, Oxycon Pro, version 4.67.01). Subsequently the exercise test was performed using an ergometric bicycle (Monark 939E) where the load was gradually increased. The rate of workload progression was determined by the age of the participant. We used 3 different protocols; participants aged 18-45 years old received a workload progression of 30 W/min, participants aged 45-55 years old a progression of 25 W/min and participants above 55 years old received a progression of 20 W/min. During the exercise breathing gases, transcutaneous oxygen and ECG-tracing were continuously analyzed and systolic blood pressure was measured frequently. A physician was present at all tests and the participants were encouraged to work until full exhaustion. Respiratory exchange ratio (RER) and oxygen consumption were continuously monitored to ensure that maximum work was performed.

### Sample size

The power analysis was performed with the assumption that vitamin D supplementation would decrease the percentage fat mass with 10% and increase maximum oxygen uptake with 10% when comparing with placebo. Using this assumption, 17 subjects were required in the treatment group and placebo group, respectively to reach a power of 80% (PS: Power and Sample Size Calculation version 3.0, 2009, Department of Biostatistics, Vanderbilt University School of Medicine). To account for some degree of drop-out we included 40 subjects in this study, 20 in each group.

### Randomization

Prior to randomization the subjects were divided into two strata based on age to provide an even age distribution between the intervention group and placebo group; one stratum with subjects aged 18-45 and one stratum with subjects aged 45–70. Each participant received then a serial number that was connected to which preparation the participant received. This information was kept in a way that was only accessible to the research nurse and the scientists involved in the study; it was accessed after the study was finished. The containers for the study drug and the placebo drops were identical and the study drug and placebo looked and tasted the same. Hence both participants and those conducting the study were blinded to which preparation the participant received. The randomization was performed by the same company that supplied the placebo and intervention drug (Apotek Produktion & Laboratorier AB, Stockholm, Sweden).

### Statistical methods

IBM SPSS Statistics software version 22.0 (IBM Corp., Armonk, NY) was used for calculations and visualization. All descriptive data are expressed as mean ± SD unless otherwise stated. Differences between groups were assessed by a two-sample un-paired *t*-test or Mann-Whitney *U*-test as appropriate; chi-2 test was used for categorical variables. Paired *t*-test was used when comparing changes within each group (baseline vs. follow-up). Linear regression was used to control for the impact of the change in physical activity on the outcomes change in percentage body fat and change in maximum oxygen uptake and the prediction ability was estimated by calculating *R*^2^, the proportion of the total variation in the outcome explained by the model. The level of significance was set at a *p* value < 0.05.

## Results

### Baseline characteristics

**Fig. 1:**
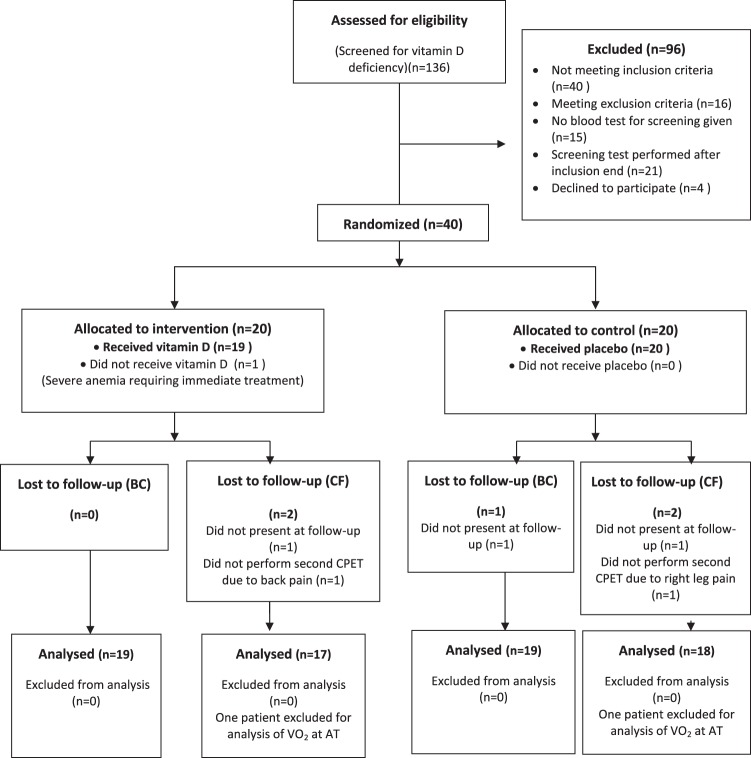
Flow Diagram of the study. BC body composition, CF cardiorespiratory fitness, CPET cardiopulmonary exercise test, VO_2_ at AT: oxygen uptake at anaerobic threshold

19 participants in the intervention group and 19 in the placebo group completed the trial and were included in the analyses concerning the outcomes fat mass and BMI, whereas for the outcomes maximum oxygen uptake, maximum load and oxygen uptake at anaerobic threshold (AT), the corresponding numbers were 17 and 18, respectively. Two additional participants were excluded from the analysis of oxygen uptake at anaerobic threshold due to technical problems with the measurement of data (Fig. 1).

Randomization was balanced with no significant differences between the groups (Table 1). Thus, there were no significant differences in age (49.8 years vs. 49.4 years, *p* = 0.92), BMI (31.5 kg/m^2^ vs. 31.2 kg/m^2^, *p* = 0.84), percentage body fat (29.7 %-units vs. 28.2 %-units, *p* = 0.47), VO_2_ max (3.02 L/min vs. 3.03 L/min, *p* = 0.62) or 25(OH)D (44.3 nmol/L vs. 44.1 nmol/L, *p* = 0.93) between groups at baseline (Table 1).

**Table 1:** Baseline characteristics of the study population

	Vitamin D (*n* = 19)		Placebo (*n* = 20)		*P* value
	Mean	SD	Mean	SD	
Age (y)	49.8	13.4	49.4	12.3	0.92
BMI (kg/m²)	31.5	5.4	31.2	3.8	0.84
Month of first vitamin D analysis	October		October		
Vitamin D (nmol/L)	44.3	8.3	44.1	6.9	0.93
Parathyroid hormone (pmol/L)	5.1	1.2	5.6	1.2	0.19
Percentage body fat (%-units)	29.7	6.2	28.2	6.3	0.47
VO_2_ max (L/min)	2.97	0.53	3.06	0.59	0.62
HDL (mmol/L)	1.2	0.3	1.0	0.3	0.06
LDL (mmol/L)	3.1	0.9	3.5	1.1	0.29
Cholesterol (mmol/L)	5.4	1.1	5.6	1.5	0.67
Glucose (mmol/L)	6.5	2.6	5.9	1.3	0.40
Insulin (mIU/L)^d^	12.6	34.2	12.4	29.4	0.88

	N	%	N	%	
Diabetes Mellitus^a^	2	11	3	15	0.68
Hyperlipidemia^a^	3	16	2	10	0.59
Hypertension^a^	6	32	7	35	0.82
Vascular events^b^	2	11	0	0	0.14
Smokers^c^	3	16	0	0	0.06

^a^ Diagnosis of diabetes mellitus, hyperlipidemia or hypertension or receiving treatment for any of the conditions

^b^ Previous stroke, TIA or myocardial infarction

^c^ Smokers and those who smoke exclusively at festive gatherings

^d^Nonsymmetrical data, median and range (max-min) displayed instead of mean and SD. Mann–Whitney *U*-test was used for comparison

Mean levels of 25(OH)D increased from 44.3 to 70.5 nmol/L in the vitamin D group and from 44.1 to 49.7 in the placebo group (*p* = 0.001 for differences between groups). Mean PTH decreased from 5.1 to 5.0 pmol/L in the vitamin D group and increased from 5.6 to 6.8 pmol/L in the placebo group (*p* = 0.06). There were two outliers in the placebo group who increased their 25(OH)D levels with 50 nmol/L and 55 nmol/L, respectively; the results regarding the statistical significance of the outcomes did not change when excluding these two participants from the analysis. Moreover, the results were consistent when including only participants with baseline 25(OH)D ≤ 50 nmol/L.

The compliance was calculated as the amount of drug taken divided by the expected amount of drug taken and was 71% for the intervention group and 77% for the placebo group.

### Body composition

The mean change in percentage body fat for the vitamin D group and the placebo group was a 0.6 ± 2.0%-units (*p* = 0.22) and a 0.1 ± 2.6%-units (*p* = 0.86) increase, respectively. The mean change in BMI in the vitamin D group was −0.2 ± 0.6 kg/m^2^ (*p* = 0.27) and in the placebo group −0.5 ± 0.9 kg/m^2^ (*p* = 0.05) (Table 2).

**Table 2:** Changes between baseline and follow-up in in the intervention and the placebo group

	Vitamin D					Placebo					Vitamin D vs. Placebo	
	Baseline		Follow up		Change^a^	Baseline		Follow up		Change^a^		
	*n*	Mean (SD)	*n*	Mean (SD)	Mean (SD)	*n*	Mean (SD)	*n*	Mean (SD)	Mean (SD)	Mean difference^b^ (95% CI)	*p*-value
25(OH)D (nmol/L)	19	44.3 (8.3)	19	70.5 (16.2)	26.3 (14.7)	19	44.2 (7.1)	19	49.8 (21.5)	5.6 (20.2)	20.7 (9.1 to 32.3)	<0.001
Weight (kg)	19	102.8 (19.4)	19	102.1 (18.9)	−0.8 (2.2)	19	100.5 (13.0)	19	99.2 (13.7)	−1.3 (3.1)	0.6 (−1.2 to 2.3)	0.53
BMI (kg/m^2^)	19	31.5 (5.4)	19	31.3 (5.2)	−0.2 (0.6)	19	31.2 (3.9)	19	30.7 (3.8)	−0.5 (0.9)	0.3 (−0.2 to 0.8)	0.26
Percentage body fat (%-units)	19	29.7 (6.2)	19	30.3 (7.5)	0.6 (2.0)	19	28.4 (6.4)	19	28.5 (6.2)	0.1 (2.6)	0.5 (−1.1 to 2.0)	0.54
Maximum VO_2_ (L/min)	17	3.02 (0.53)	17	3.04 (0.61)	0.02 (0.23)	18	3.03 (0.59)	18	3.06 (0.71)	0.03 (0.24)	−0.01 (−0.2 to 0.2)	0.90
Maximum VO_2_/kg (ml/min/kg)	17	30.0 (6.4)	17	30.4 (7.8)	0.4 (2.5)	18	31.1 (7.8)	18	31.7 (8.7)	0.6 (2.4)	−0.2 (−1.9 to 1.5)	0.80
Maximum load (Watt)	17	242.8 (52.3)	17	246.0 (54.8)	3.2 (19.0)	18	248.7 (58.9)	18	252.7 (56.3)	4.0 (11.7)	−0.8 (-11.5 to 10.0)	0.89
Maximum heart rate (bpm)	17	167.6 (15.8)	17	164.3 (18.3)	−3.4 (11.7)	18	162.5 (17.7)	18	164.2 (19.2)	1.7 (7.9)	−5.1 (−11.9 to 1.8)	0.14
VO_2_ at AT (L/min)	16	1.66 (0.41)	16	1.79 (0.49)	0.13 (0.26)	17	1.78 (0.39)	17	1.78 (0.45)	0.0 (0.27)	0.13 (−0.05 to 0.33)	0.14
Reported change in physical activity	Vitamin D *n* = 12					Placebo *n* = 14						0.02^c^
Less activity post, *n* (%)	0 (0%)					6 (43%)						
No change, *n* (%)	8 (67%)					7 (50%)						
More activity post, *n* (%)	4 (33%)					1 (7%)						

^a^Change = Follow up minus Baseline

^b^Between group differences were compared using a two-sample *t*-test

^c^Chi-2 test was used for the variable “reported change in physical activity”

**Fig. 2:**
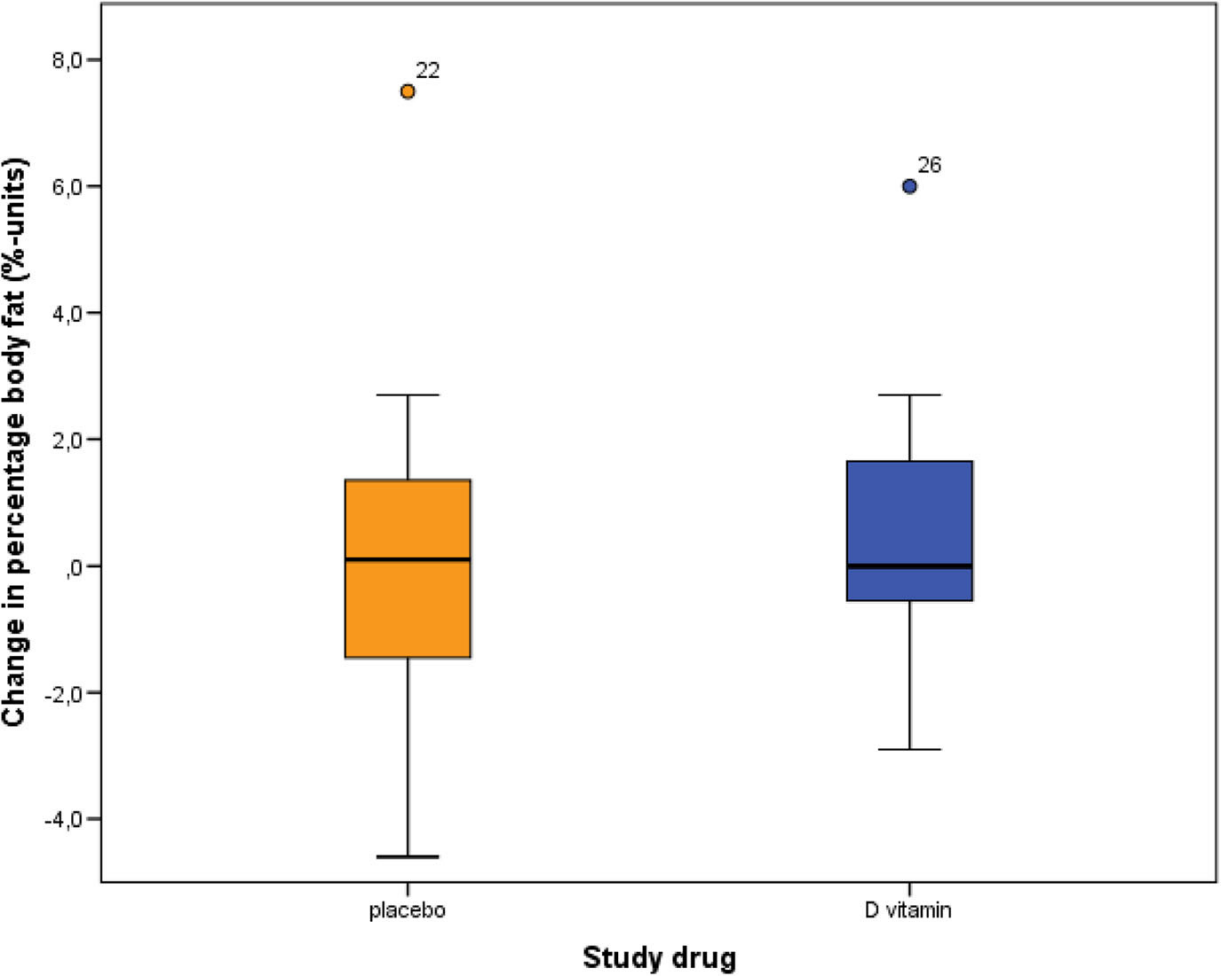
Change in percentage body fat between baseline and follow-up in the placebo and the intervention group. Mean difference 0.5 %-units (95% CI −1.1–2.0), *p* = 0.54

There was no statistically significant difference between the intervention and the placebo group regarding change in percentage body fat (mean difference 0.5 %-units, 95% CI −1.1–2.0, *p* = 0.54) or BMI (mean difference 0.3 kg/m^2^, 95% CI −0.2–0.8, *p* = 0.26) (Table 2, Fig. 2). Moreover, no statistically significant difference was observed between the groups regarding change in any of the metabolic laboratory values (cholesterol, high-density lipoprotein, low-density lipoprotein, triglycerides, fasting plasma glucose, and insulin).

In the placebo group, 43 % of the participants reported that they had decreased their physical activity, 7% had increased and in 50 % there was no difference. In the intervention group the corresponding figures were 0%, 33%, and 67%, respectively. The difference in change in physical activity between the groups was statistically significant (*p* = 0.02).

The multiple linear regression analysis showed no significant impact of change in exercise or change in vitamin D on change in percentage body fat (*R*^2^ 0.166, *p* = 0.11).

### Cardiorespiratory fitness

Attainment of VO_2max_ was affirmed; all subjects met at least 2 of the 3 following criteria: plateau of oxygen uptake, volitional exhaustion or a calculated respiratory exchange ratio (RER) > 1.10. Mean RER at maximal work was 1.21 ± 0.06 at baseline and 1.22 ± 0.06 at follow up. The mean change in maximum oxygen uptake was a 0.023 ± 0.23 L/min increase in the intervention group (*p* = 0.69) and a 0.033 ± 0.24 L/min increase in the control group (*p* = 0.56). The mean change in maximum load was a 3.2 ± 19 W increase in the intervention group (*p* = 0.49) and a 4.0 ± 11.7 W increase in the control group (*p* = 0.17). The mean change in oxygen uptake at AT was a 0.13 ± 0.26 L/min increase in the intervention group (*p* = 0.05) and a 0.005 ± 0.27 L/min decrease in the control group (*p* = 0.94).

**Fig. 3:**
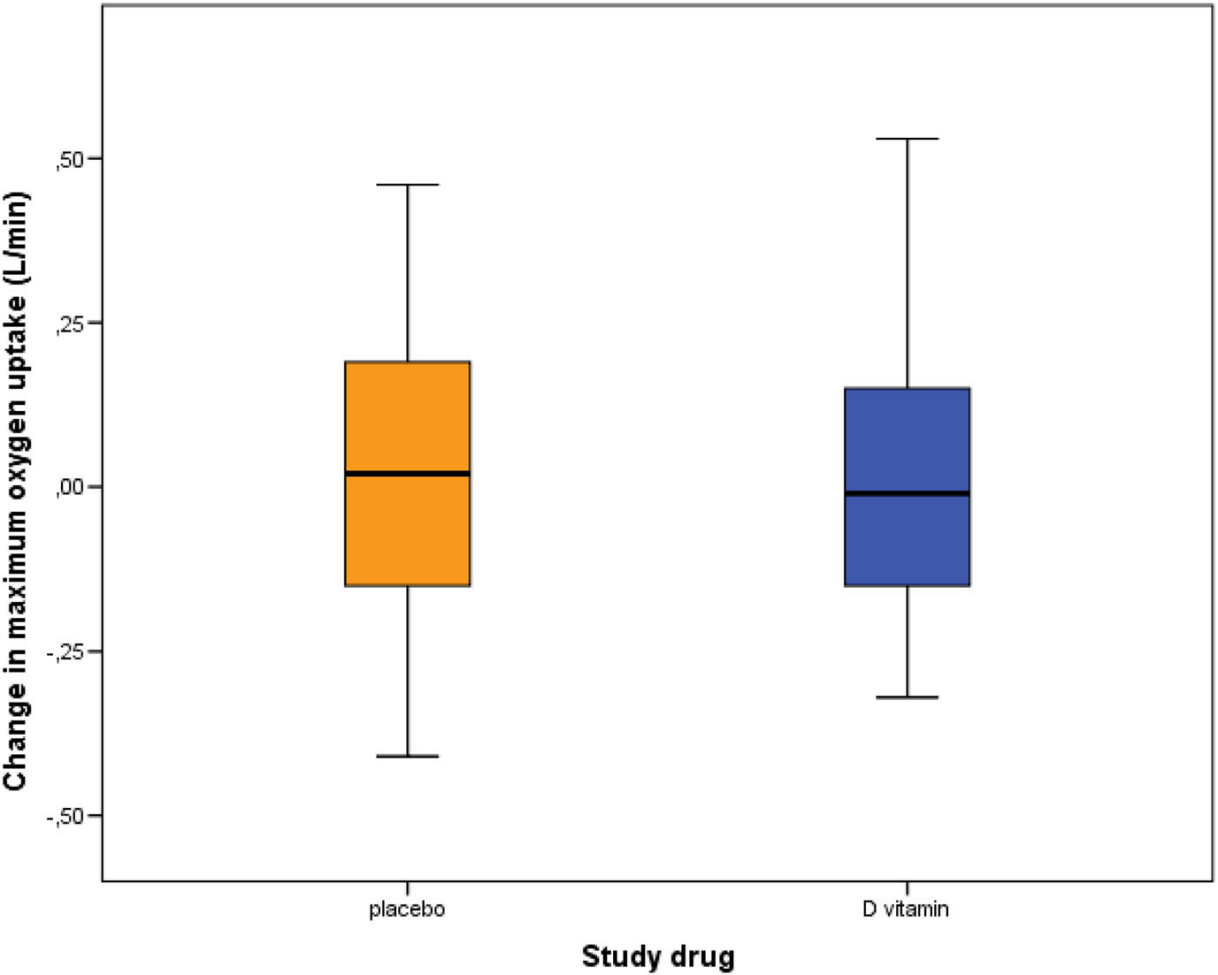
Change in maximum oxygen uptake between baseline and follow-up in the placebo and the intervention group. Mean difference −0.01 L/min (95% CI −0.2–0.2), *p* = 0.90

There was no statistically significant difference between the intervention and the placebo group concerning change in the outcomes maximum oxygen uptake (mean difference −0.01 L/min, 95% CI −0.2–0.2, *p* = 0.90) (Fig. 3), maximum load (mean difference -0.8 W, 95% CI −11.5–10.0, *p* = 0.89) and oxygen uptake at AT (mean difference 0.13 L/min, 95% CI −0.05–0.33, *p* = 0.14) (Table 2).

The multiple linear regression analysis showed no significant impact of change in exercise or change in vitamin D on change in maximum oxygen uptake (*R*^2^ 0.077, *p* = 0.41).

### Adverse events

There were no reports of side effects or adverse events and no subjects developed hypercalcemia during the study.

## Discussion

In this randomized, double-blind, placebo-controlled trial we investigated the effects of vitamin D supplementation in overweight men with vitamin D deficiency at baseline. The 6-month treatment raised the plasma 25(OH)D levels significantly but had no effect on body weight, percentage body fat or cardiorespiratory fitness, measured as maximum oxygen uptake. Neither did we find any effect of vitamin D supplementation in any of the metabolic laboratory values. The results were consistent when including only participants with baseline 25(OH)D ≤ 50 nmol/L. We observed an increase in plasma 25(OH)D even in the placebo group which can be attributed both to there being two outliers in this group and to seasonal variation, although we tried to avoid the latter. The change in the placebo group could conceal the effects of treatment, but our results did not change when excluding these two participants from the analysis. Moreover, we noticed a statistically significant difference in change in physical activity between the groups, which could be a potential confounding factor; however, multiple linear regression showed no significant impact of change in exercise or change in vitamin D on change in maximum oxygen uptake or in percentage body fat.

Varying guidelines exist for defining optimal serum concentrations of 25(OH)D in humans [33, 34]. According to the currently available RCTs, no conclusion can be drawn with regard to optimal vitamin D intake or 25(OH)D levels for extraskeletal health [35]. However, based on association studies, the greatest risk of several major diseases including cardiovascular and metabolic diseases is found in subjects with 25(OH)D levels below 50 nmol/L. Our target was therefore to assure 25(OH)D levels above 50 nmol/L in the treatment group, which was achieved; 25(OH)D increased from 44.3 nmol/L at baseline to 70.5 nmol/L at follow-up.

A number of recently published studies supports our results; in a randomized, double-blind trial (52 subjects–15 males, aged 18–50 years, BMI > 30 kg/m^2^, 25(OH)D < 50 nmol/L randomized to receive 7000 IU vitamin D daily or placebo in 26 weeks) Wamberg et al found that treatment did not change body fat, BMI, insulin resistance or plasma lipids [18]. However, 17% of the randomized subjects were lost to follow-up which might have limited the ability to detect significant differences between groups. Sneve et al found that treatment of 445 overweight or obese adults with 20,000 IU vitamin D twice weekly for 12 months did not lead to weight reduction [36]. Salehpour et al. found no significant change in body weight or BMI but a significant decrease in body fat mass in the vitamin D group in their randomized RCT where 77 healthy overweight and obese women were randomized to receive either 1000 IU vitamin D per day or placebo for 12 weeks [37]. A large observational study comparing laboratory test results from 10,8711 subjects examined the association between plasma lipids and 25(OH)D and found a significant cross-sectional association between 25(OH)D levels and components of the lipid panel. However, the longitudinal analysis showed that increasing 25(OH)D levels from the deficient to optimal range was only associated with small and clinically minimal effects on lipid profile [38]. Gregory et al investigated the relationships between changes in 25(OH)D and changes in aerobic fitness (VO_2max_) over 6 months in 213 healthy adults and found that neither baseline 25(OH)D levels nor changes in 25(OH)D levels predicted changes in cardiorespiratory fitness. The authors suggest that the study duration might justify the lack of association since substantial changes in 25(OH)D or VO_2max_ are unlikely to occur over 6 months without a controlled intervention [39].

Thus, there is a big discrepancy between cross-sectional studies associating low vitamin D levels to increased BMI, increased percentage body fat, impaired cardiorespiratory fitness, and various other metabolic complications and the lack of effects of vitamin D supplementation in clinical trials. However, most of these trials have small sample sizes and short duration and might not have enough statistical power to detect minor effects of vitamin D supplementation. An alternative explanation for this observation could be that high 25(OH)D levels are only a marker of good health and a healthier life style; because obese individuals more often have various health problems, they will also have low 25(OH)D levels. The mounting knowledge about the multiple effects of 1,25(OH)_2_D_3_ in experimental studies implies, however, that vitamin D supplementation might have beneficial clinical effects.

Indeed, 1,25(OH)_2_D_3_ regulates adipogenesis at various levels of the entire differentiation process; in mouse 3T3-L1 preadipocytes 1,25(OH)_2_D_3_ inhibited adipogenesis in early stages of differentiation by acting on multiple targets suppressing master transcription factors [14, 40]. Moreover, vitamin D receptor (VDR), in the presence of 1,25(OH)_2_D_3_, downregulated adipocyte promoting transcription factors at a critical stage of differentiation and interestingly, in the absence of 1,25(OH)_2_D_3_, VDR knockdown prevented lipid accumulation in adipocytes [41]. Finally, 1,25(OH)_2_D_3_ has been shown to significantly reduce inflammation in the adipose tissue (inflammation is common in obesity where adipose tissue undergoes hypertrophic enlargement which results in an imbalanced blood flow) [14].

Insulin resistance is a common feature of obesity and predisposes to the development of type 2 diabetes mellitus [11]. Human cross-sectional studies have shown that low levels of vitamin D are associated with hyperglycemia [42, 43], with hyperinsulinemia [44], with decreased beta-cell function and with measures of insulin resistance [45]. Vitamin D has been suggested to have beneficial effects on both insulin secretion and insulin sensitivity [12]. In a recently published review of randomized controlled trials [46], Zuk et al. reported that fasting plasma glucose levels increased less in the vitamin D group than placebo with sufficient baseline mean 25(OH)D concentrations and decreased with insufficient baseline mean 25(OH)D concentrations following vitamin D repletion, supporting the inverse association between vitamin D and glucose measure among overweight/obese individuals.

CRF is determined primarily by maximal cardiac output and maximal arteriovenous O_2_ difference and thus potential mechanisms of how vitamin D may affect CRF should include alterations in maximal heart rate and maximal stroke volume, as well as in skeletal characteristics. As far as muscle mass and strength is concerned, binding of 1,25(OH)_2_D_3_ to VDR in human skeletal muscle up-regulates gene transcription of mRNA and increases muscle protein synthesis [47] while abnormal muscle metabolism and synthesis have been demonstrated in VDR knockout mice [48]. Moreover, it is suggested that an increased transport of Ca^+^ and P into muscle cells occurs following the binding of 1,25(OH)_2_D_3_ to VDR, allowing for more efficient ATP production and contractility of the muscle [49]. It should be noted that we did not measure 1,25(OH)_2_D_3_ in this study and it is possible that the blood concentration of this metabolite remained unchanged despite the significant change in 25(OH)D as the biologically active form of vitamin D is tightly regulated.

The strength of our study lies in the double-blind randomized controlled design, which allows for causative conclusions. A limitation of our study is that we used BIA in order to assess body composition and not dual X-Ray absorptiometry (DXA) which is considered the gold standard method. However, BIA is also a validated and reliable method for measurement of body composition in obese adults. Second, our relatively small sample size could be another limitation; the sample size in this study was calculated to detect a change of 10% or more in percentage body fat and in VO_2max_ which might have been too much. Thus clinical trials with a larger size are warranted. Finally, the short duration of vitamin D supplementation in our study may be insufficient to counteract years of exposure to low vitamin D levels. Obesity and its complications develop over years and treatment for six months might be a too short period to see an improvement in body composition or cardiorespiratory fitness.

In conclusion we found that treatment with 2000 IU vitamin D for 6 months does not impact body composition or maximum oxygen uptake in overweight men with vitamin D deficiency. Clinical trials with larger size and longer duration are warranted.
